# Phenotypic and genotypic characteristics of macrolide, lacosamide, and streptogramin resistance in clinically resistant *Streptococci* and their correlation with reduced biocide susceptibility

**DOI:** 10.1186/s12916-025-04097-9

**Published:** 2025-05-13

**Authors:** Safaa A. Abdel-Karim, Fathy M. Serry, Eman M. Elmasry, Wael A. H. Hegazy

**Affiliations:** Department of Microbiology and Immunology, Faculty of Pharmacy, Zagazig University, Zagazig, 44519 Egypt

**Keywords:** *Streptococci* spp., Macrolides, Lincomsamides, Streptogramins, MLS Phenotypes, MLS Genotyping, Biocides

## Abstract

**Background:**

Gram-positive *Streptococci* is a huge group of different species that are classified based on its hemolytic effect besides the C-substance in the cell wall. This study focuses on the investigation of the prevalence and genetic basis of resistance to macrolides, lincosamides, and streptogramins (MLS) in α- and β-hemolytic *Streptococci*.

**Methods:**

Streptococcal isolates were identified and their resistance was assessed to MLS antibiotics through phenotypic analysis and genotypic screening of resistance genes. Isolates were also tested for susceptibility to antiseptics/disinfectants. The correlation between high MLS antibiotic resistance and reduced susceptibility to biocides was assessed. Efflux pump activity in the most resistant isolates (to both MLS antibiotics and biocides) was investigated.

**Results:**

The susceptibility testing indicates an increasing resistance to MLS, particularly macrolides (erythromycin, azithromycin, and clarithromycin) and lincomycin. By screening the resistance, the most predominant phenotype is the constitutive (cMLS) one, while the *erm* genes, particularly *ermB*, are the most detected genotype. Furthermore, the esterase-encoding gene *ereA* is widely distributed in the streptococcal isolates. By evaluating the minimum inhibitory concentrations (MICs) to different biocides, there was a strong relation between the increased MIC values to both MLS antibiotics and tested biocides. This can be attributed mainly to the transferable *ermB* gene and the enhanced bacterial efflux.

**Conclusions:**

A significant correlation exists between reduced biocide susceptibility and resistance to MLS antibiotics. Elevated efflux pump activity in MLS-resistant isolates suggests efflux mechanisms may contribute to dual resistance to antibiotics and biocides. However, cross-resistance is primarily driven by the horizontally transferable ermB gene, which confers resistance by targeting the 50S ribosomal subunit.

**Supplementary Information:**

The online version contains supplementary material available at 10.1186/s12916-025-04097-9.

## Background

The *Streptococcus* genus comprises diverse Gram-positive cocci bacteria with notable medical importance. These bacteria are capable of inducing a spectrum of diseases, ranging from subacute to acute or chronic. Some notable human ailments attributed to *Streptococci* include rheumatic heart disease, scarlet fever, pneumococcal pneumonia, and glomerulonephritis [1]. *Streptococci* are classified based on the hemolytic pattern exhibited on blood agar plates, which categorizes them into alpha-hemolytic, beta-hemolytic, and gamma-hemolytic groups. Another classification criterion is based on Lancefield grouping, which involves the identification of specific polysaccharide antigens in the bacterial cell wall [2].

Among the most clinically important *Streptococci* spp., *S. pyogenes* is commonly referred to as Group A streptococci (GAS). *S. pyogenes* can be classified into over 100 M-serotypes or *emm* types, determined by their M proteins present on the cell surface. These proteins contribute to their virulence by impeding phagocytosis. *S. pyogenes* represents a significant source of infections among children in both outpatient and hospital environments. They are known to cause various conditions such as pharyngitis, impetigo, ecthyma, erysipelas, and cellulitis, along with severe invasive diseases like necrotizing fasciitis and streptococcal toxic shock syndrome [3, 4]. Another medically important β-hemolytic streptococci, *S. agalactiae* (group B) causes a wide range of invasive diseases among both infants and adults. In adults, bacteremia is one of the most common syndromes resulting from invasive *S. agalactiae* that can lead to seeding of the cardiac valves and endocarditis [5–7].

*S. pneumoniae* is an extracellular, opportunistic α-hemolytic streptococcal spp. that colonizes the upper respiratory tract mucosal surfaces. *S. pneumoniae is* present in the nasopharynx*,* as between 27 and 65% of children and fewer than 10% of adults are *S. pneumoniae* carriers [8]. *S. pneumoniae* dissemination to the bloodstream and lower respiratory tract could lead to invasive inflammatory diseases including otitis media, community-acquired pneumonia, sepsis, and meningitis [9, 10]. Viridans streptococci represent another group of clinically significant *Streptococci* spp. Among the prominent members of the viridans streptococci, which are typically commensal, *S. sanguis* and *S. mutans* cause dental caries, *S. mitis* is associated with meningitis, bacteremia, pneumonia, and periodontal disease, and *S. milleri* is associated with purulent infections in both children and adults [11, 12].

Macrolide and lincosamide antibiotics, while chemically dissimilar, share similar modes of action. These antibiotics were considered a substitute for penicillins and cephalosporins for a long period. Nonetheless, the emergence of macrolide resistance has restricted the application of these antibiotics to specific diseases [13–17]. Natural macrolides are comprised of a 14–16 membered lactone ring linked to two amino or neutral sugars. More recent semisynthetic macrolides were developed with modifications on the lactone ring, enhancing their antimicrobial efficacy and acid stability [17, 18]. Lincosamides consist of naturally occurring lincomycin and clindamycin, the semi-synthetic derivative of lincomycin. Although lacking the characteristic macrolide lactone ring, lincosamides operate through a similar mechanism, targeting the 50S bacterial sub-ribosomal unit [13, 17]. The bacterial protein synthesis inhibition by macrolides and lincosamides involves their reversible binding to the bacterial ribosome 50S subunit. This action stimulates the dissociation of the peptidyl-tRNA from the ribosome during the elongation process, ultimately leading to chain termination [18]. Another class of antibiotics that binds reversibly to the bacterial ribosomal 50S subunit is the streptogramins, which are categorized into two groups, streptogramin A and B, which synergistically combine to inhibit bacterial protein synthesis [19].

The incidence of macrolide, lincosamide, and streptogramin (MLS) resistance is rising between Gram-positive clinical isolates. The diversity of resistance mechanisms associated with these drugs leads to a range of resistance phenotypes [13, 20]. In Gram-positive bacteria, three distinct mechanisms of acquired MLS resistance have been identified: target-site modification through methylation or mutation of 23S rRNA, efflux of the antibiotic, and enzymatic inactivation. The most prevalent and clinically significant resistance mechanisms are the methylation of the 23S rRNA ribosomal subunit and the efflux of the drug [13, 18, 21]. While these modifications impart broad-spectrum resistance to macrolides and lincosamides, enzymatic modifications impact only structurally related antibiotics [13, 21].

The biocides utilization in various consumer products such as plastics, household items, cosmetics, and more has been identified as a risk factor leading to the development of antimicrobial resistance [22]. Disinfectant biocides typically do not promote cross-resistance to antibiotics. However, in vitro cultures have demonstrated the isolation of bacteria that are resistant or more tolerant following exposure to suboptimal or sublethal levels of biocides [23]. The primary objective of this study was to identify prevalent resistance patterns, resistance phenotypes, and predominant resistance genes related to macrolides and lincosamides among local clinical *Streptococci* isolates. Additionally, the study aimed to examine the potential correlation between resistance to MLS antibiotics and the susceptibility of streptococci to commonly used biocides.

## Methods

### Bacterial specimens collection and identification

In this study, a total of 825 clinical samples was gathered from Zagazig University Hospitals, Zagazig, Egypt. The acquisition of patients'consent for microbiological examinations adhered to the hospital's established protocols and was overseen by the hospital administration department. This process fully aligned with the principles outlined in the Helsinki Declaration, ensuring the patients’ participation posed no risk, danger, or burden to them. Importantly, the clinical specimens were procured from microbiological laboratories without any direct patient contact. Standard microbiological techniques were employed for the subsequent identification of the isolates [24].

### Determination susceptibility to MLS antibiotics

All the identified streptococcal isolates underwent susceptibility testing against MLS antibiotics using the disk diffusion technique, following the guidelines outlined by the Clinical and Laboratory Standards Institute (CLSI) in 2021 [15]. Additionally, the minimum inhibitory concentrations (MICs) of the tested biocides or antibiotics were determined using the agar dilution method, also in accordance with CLSI 2015 guidelines [14, 25]. Furthermore, MIC_50_ and MIC_90_ values were calculated that represent the concentrations at which 50% and 90% of the isolates were inhibited, respectively. This calculation involved determining the median for MIC_50_ and the 90 th percentile for MIC_90_ [26, 27].

### Recognition of MLS resistance phenotypes

The triple disk diffusion test was conducted according to Novotna et al. [27, 28]. Overnight cultures of tested isolates in tryptone soya broth (TSB) were standardized equivalent to a 0.5 McFarland standard. These suspensions were then evenly spread on the Müeller-Hinton (MH) agar plates, and clindamycin (2 µg), erythromycin (15 µg), and lincomycin (2 µg) disks were positioned close to each other. The inhibition zones were examined after overnight incubation at 37 °C. The interpretation of the inhibition zones was as follows: (i) If the isolates were resistant to the three antibiotics and there were no inhibition zones, it was classified as constitutive resistance (cMLS) phenotype. (ii) Any flattening or alteration in the shape of the clindamycin zone indicated an inducible resistance (iMLS) phenotype. (iii) Resistance to lincomycin while being sensitive to erythromycin and clindamycin was considered an L-phenotype. (iv) Isolates sensitive to lincomycin and clindamycin but exhibited resistance to erythromycin were categorized as M-phenotypes.

### Recognition of MLS resistance genotypes

The detection of the genes that are involved in the MLS resistance was accomplished by multiplex PCR. The DNA extraction kit purchased from Qiagen (Düsseldorf, Germany) was used to extract bacterial DNA [29], and kept at – 80 °C [29, 30]. The multiplex PCR was performed for the gene that is involved in erythromycin ribosome methylase (*erm*), efflux (*msr* and *mef*) and MLS-modifying enzymes *ereA*, *lnuA*, and *mphC*. The used primers were listed in previous studies (Table 1) [27, 31–36].

**Table 1:** List of primers

Gene	Primer	Primer sequence (5′−3′)	References
***erm*A**	**F**	AAGCGGTAAACCCCTCTGA	[31]
	**R**	TTCGCAAATCCCTTCTCAAC	
***erm*B**	**F**	CTATCTGATTGTTGAAGAAGGATT	[36]
	**R**	GTTTACTCTTGGTTTAGGATGAAA	
***erm*C**	**F**	AATCGTCAATTCCTGCATGT	[31]
	**R**	TAATCGTGGAATACGGGTTTG	
***msr*A**	**F**	TCCAATCATTGCACAAAATC	[36]
	**R**	AATTCCCTCTATTTGGTGGT	
***mef*A**	**F**	CGTAGCATTGGAACAGC	[32]
	**R**	TGCCGTAGTACAGCCAT	
***mef*E**	**F**	CGTAGCATTGGAACAGC	[32]
	**R**	TCGAAGCCCCCTAATCTT	
***ere*A**	**F**	AACACCCTGAACCCAAGGGACG	[33]
	**R**	CTTCACATCCGGATTCGCTCGA	
***lnu*A**	**F**	GGTGGCTGGGGGGTAGATGTATTAACTGG	[35]
	**R**	GCTTCTTTTGAAATACATGGTATTTTTCGATC	
***mph*C**	**F**	ATGACTCGACATAATGAAAT	[34]
	**R**	CTACTCTTTCATACCTAACTC	

### Evaluation of the efflux in the resistant isolates

The efflux efficiency was quantitatively assayed by measuring fluorescence of the expelled ethidium bromide (EtBr) (Sigma-Aldrich, St. Louis, MO, USA) in the presence of glucose as efflux activator and di-nitro phenol (DNP) (Sigma-Aldrich, St. Louis, MO, USA) as inhibitor [37]. The highly resistant isolates with high MIC > MIC_50_ to both antibiotics and biocides were nominated to be compared with those which showed lower MIC < MIC_50_ to both antibiotics and biocides. The broth microdilution method was used to determine the MICs of the selected isolates against EtBr and DNP according to CLSI, 2021 [38]. To exclude the influence of DNP on the cellular viability, it was used at 1/4 MIC concentrations, and hence the MIC of EtBr in the presence of DNP at 1/4 MIC was determined.

The optical densities of tested isolates were adjusted to OD600 of 0.6, the bacterial cells were collected by centrifugation, and washed with phosphate buffer saline (PBS). The maximum accumulation optimal conditions of EtBr in the absence or presence of 0.4% glucose were initially detected. For each assay, 50 µl of the bacterial suspensions were combined with an equal volume of varying concentrations of EtBr in the absence or presence of glucose, and the fluorescence levels were measured. The real-time monitoring of EtBr accumulation and extrusion was performed. All readings were taken at the excitation 530 nm and emission 585 nm wavelengths specific to EtBr. The fluorescence data were collected at intervals of 1 min over a period of 1 h at a temperature of 25 °C. The experiments were replicated three times, and the results were averaged. Following the establishment of the EtBr accumulation proper conditions, the impact of DNP was investigated on its accumulation in the presence or absence of 0.4% glucose, and the fluorescence levels were measured. To quantify the efflux activity of the tested isolates, the bacteria were mixed with EtBr (at sub-MIC) for 1 h by centrifugation at room temperature. The collected pellets were resuspended with cold PBS. Equal volumes of the resuspended bacterial cells were added to microtiter plate wells provided with PBS with or without 0.4% glucose, or with DNP in concentrations that promote the highest EtBr accumulation. The fluorescence was measured at a temperature of 37 °C, as previously outlined. The EtBr efflux is quantified in relative fluorescence (RF) units, which were determined by comparing the fluorescence in the absence or presence of glucose with the fluorescence of samples in the absence of glucose and the presence of DNP as a negative efflux control using theformula:

$$\text{RF} \, = \frac{{\left( {\text{PBS {-} glucose}} \right){ - }\left( {\text{PBS + glucose}} \right)}}{{\text{PBS {-} glucose + DNP}}}$$

## Results

### Isolation and identification of streptococcal isolates

**Fig. 1:**
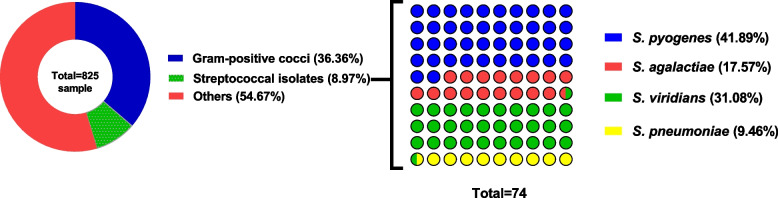
Occurrence of streptococcal spp. From 825 clinical isolates, 36.4% were Gram-positive cocci that were further identified to approximately 9% streptococcal spp. Additionally, the *Streptococcal* spp. was presumptively identified into *S. pyogenes* (41.9%), *S. agalactiae* (17.6%), *S. viridians* (31.1%) and *S. pneumoniae* (9.5%)

Three hundred and seventy-four Gram-positive cocci isolates were obtained from collected 825 clinical isolates. Seventy-four were identified as *Streptococci* (19.8%) based on microscopic examination and biochemical identification (Table 2). The isolates were identified into 31 *S. pyogenes*, 13 *S. agalactiae*, 23 *S. viridians*, and 7 *S. pneumoniae* (Fig. 1). The source of streptococcal isolates was shown (Additional file 1: Table S1).

**Table 2:** Biochemical identification of streptococcal spp

Biochemical test	*S. pyogenes*	*S. agalactiae*	*S. viridans*	*S. pneumoniae*
Catalase	−	−	−	−
Motility	Non-motile	Non-motile	Non-motile	Non-motile
Growth in 6.5% NaCl	−	−	−	−
Growth at 10 °C	−	−	−	−
Growth at 45 °C	−	−	−	−
Growth on MacConkey agar	−	−	−	−
Mannitol Fermentation	−	−	Variable	−
Blood hemolysis	β-hemolysis	β-hemolysis	α-hemolysis	α-hemolysis
Sensitivity to bacitracin	Sensitive	Resistant	−	−
Sensitivity to optochin	−	−	Resistant	Sensitive
PYR test	+	−	na	na
CAMP test	−	+	na	na
Bile solubility	na	na	Insoluble	Soluble
Inulin fermentation	na	na	−	+

### MLS susceptibility

**Fig. 2:**
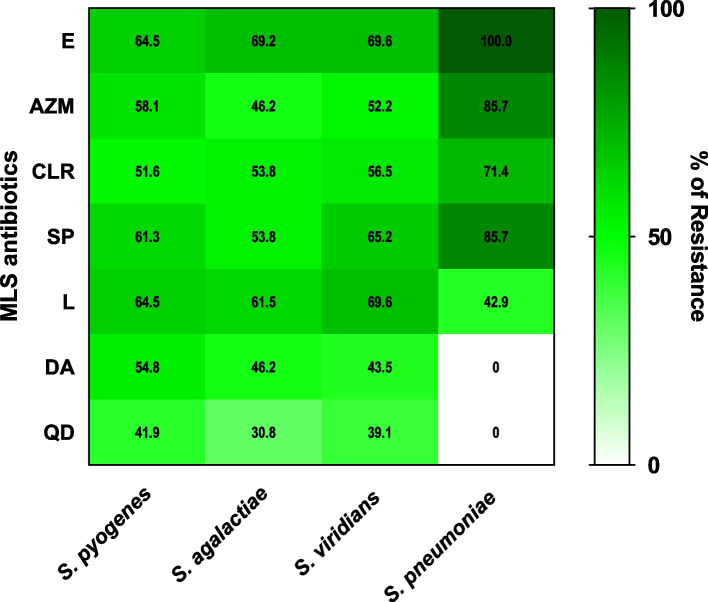
The streptococcal resistance rates to the MLS antibiotics. E = Erythromycin, CLR = Clarithromycin, AZM = Azithromycin, L = Lincomycin, SP = Spiramycin, QD = Quinupristin/Dalfopristin, and DA = Clindamycin

The susceptibility of streptococcal isolates was tested to erythromycin (E), clarithromycin (CLR), azithromycin (AZM), lincomycin (L), spiramycin (SP), quinupristin/dalfopristin (QD), and clindamycin (DA) by disk diffusion method. The resistance percentages to different MLS antibiotics are presented in Fig. 2, and detailed (Additional file 1: Table S2).

To assess the association between the type of antibiotic or microorganism and the resistance, a generalized mixed model was employed using R Project for Statistical Computing (v. 4.3.0). The results were presented as odds ratio and 95% CI (Additional file 1: Table S3). The resistance of streptococcal isolates to AZM, CLR, SP, L, DA, and QD were reduced by 72%, 75%, 41%, 41%, 89%, and 93%, respectively, compared with the resistance to erythromycin. However, the highest and the lowest percentages of resistance were observed in *S. pneumoniae* to erythromycin and clindamycin, respectively; the differences in the resistance of all streptococcal isolates to different MLS antibiotics were not statistically significant.

### MLS resistance phenotypes

**Fig. 3:**
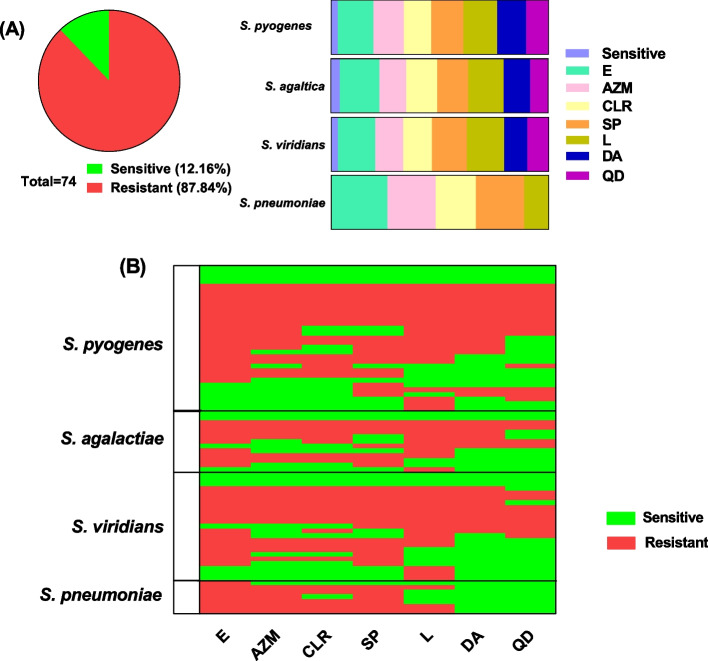
The MLS resistance patterns. **A** Percentage of the resistant and sensitive isolates. **B** Heat map represents the resistance patterns. E = Erythromycin, CLR = Clarithromycin, AZM = Azithromycin, L = Lincomycin, SP = Spiramycin, QD = Quinupristin/Dalfopristin, and DA = Clindamycin

Among 74 streptococcal isolates, 65 isolates (87.8%) showed resistance to MLS antibiotics (Fig. 3A). The resistant isolates were 27 (87.1%) *S. pyogenes*, 11 (84.6%) *S. agalactiae*, 20 (87%) *S. viridans*, and 7 (100) *S. pneumoniae*. These resistant isolates were subjected to further investigation. The resistance patterns were represented in Fig. 3B; furthermore, the resistance patterns were detailed (Additional file 1: Table S4).

The inhibition zones formed around the clindamycin, erythromycin, and lincomycin disks were observed to ascertain the resistance phenotype, as previously shown [27]. The MLS resistance phenotypes are summarized in Table 3.

**Table 3:** MLS phenotypes

Isolates	Resistance phenotype				Total
	**cMLS**	**iMLS**	**M**	**L**	
***S. pyogenes***	**17 (63%)**	**2 (7.4%)**	**5 (18.5%)**	**3 (11.1%)**	**27**
***S. agalactiae***	**6 (54.5%)**	**2 (18.2%)**	**2 (18.2%)**	**1 (9.1%)**	**11**
***S. viridians***	**10 (50%)**	**3 (15%)**	**4 (20%)**	**3 (15%)**	**20**
***S. pneumoniae***	**0 (0%)**	**3 (42.9%)**	**4 (57.1%)**	**0 (0%)**	**7**
**Total**	**33 (50.8%)**	**10 (15.4%)**	**15 (23.1%)**	**7 (10.8%)**	**65**

*cMLS *constitutive macrolides, lincosamides, and streptogramin B resistance phenotype; *iMLS *inducible macrolide, lincosamide, and streptogramin resistance phenotype; *M *macrolides and streptogramin B or macrolides resistance phenotype; *L *lincosamides inactivation resistance phenotype; antibiotic discs *E *erythromycin 15 µg; *DA *clindamycin 2 µg; *L *lincomycin 2 µg

### Genotyping of MLS resistance

**Fig. 4:**
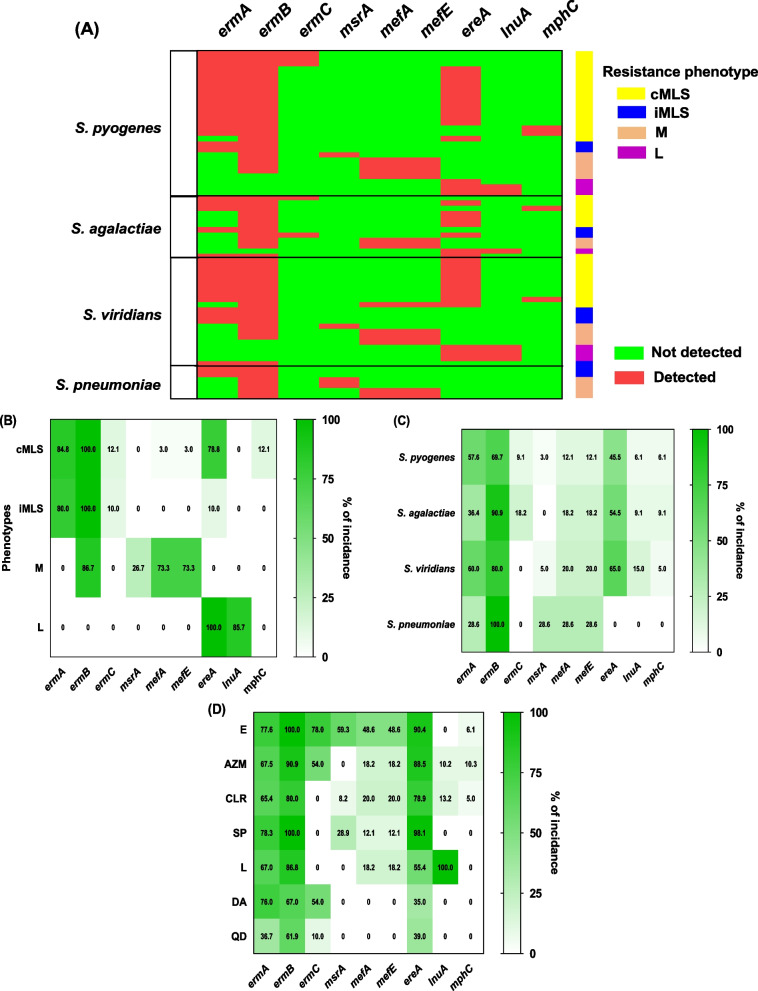
MLS resistance genotypes. **A** The distribution of the MLS resistance genes in the resistant isolates. **A** Heat maps represent the MLS resistance genes percentages in different **B** phenotypes and **C** *Streptococci* spp., and **D** MLS antibiotics. E = Erythromycin, CLR = Clarithromycin, AZM = Azithromycin, L = Lincomycin, SP = Spiramycin, QD = Quinupristin/Dalfopristin and DA = Clindamycin

The current results identified all examined resistance encoding genes within the resistant isolates, as illustrated in (Fig. 4) and detailed in Additional file 1: Table S5. The most prevalent gene, *ermB*, was detected in all cMLS-phenotype isolates, resistant *S. pneumoniae*, and other erythromycin-resistant strains. In contrast, *ermB* was absent in all L-phenotype isolates. The least detected gene, *ermC*, was not identified in M- or L-phenotypes and was also absent in α-hemolytic streptococci. Regarding resistance genes encoding modifying enzymes, the current findings revealed that *ereA* (associated with hydrolyzing enzymes) was the most prevalent, significantly outnumbering *lunA* and *mphC*. The *ereA* gene was predominantly detected in both cMLS- and L-phenotype isolates. In contrast, *lunA* was exclusively identified in L-phenotypes, while *mphC* was restricted to cMLS-phenotypes. Additionally, the efflux-encoding genes were detected in nearly all *Streptococcus* species, with the exception of *msrA*, which was absent in *S. agalactiae*. Notably, these genes were more prevalent in M-phenotype isolates.

### The increase of MLS and biocides MICs

The agar dilution method was employed to detect MICs of MLS antibiotics in the resistant isolates as shown (Additional file 1: Table S6). The MICs to MLS antibiotics ranged between 0.125 and 1024 µg/mL. It is observed that the lowest MIC required to inhibit 50% of the bacterial growth was detected with lincomycin and quinupristin/dalfopristin. Moreover, the MICs of the resistant isolates were detected against thiomersal, chlorocresol, cetrimide, triclosan, povidone-iodine, and glutaraldehyde that represent different biocides (Additional file 1: Table S7). The highest MIC range was observed with povidone-iodine.

### The relationship between reduced biocide susceptibility and MLS resistance

**Fig. 5:**
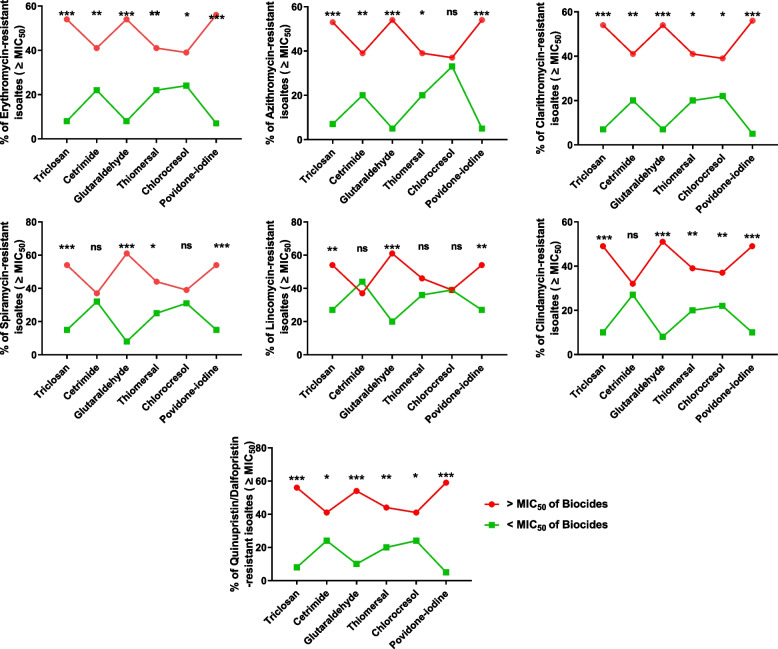
The percentages of the reduced susceptibility to biocides in the MLS-resistant isolates that have MIC ≥ MIC_50_. Chi-square test was employed to assess the statistical differences between the percentages of MLS-resistant isolates in high and low levels of MICs to biocide. ns: *p* > 0.05, *: *p* ≤ 0.05, **: *p* ≤ 0.01, ***: *p* ≤ 0.001

The Streptococcal isolates were regarded as either relatively less susceptible or susceptible to biocides and MLS antibiotics based on their MIC_50_ values [39]. Reduced susceptibility was attributed to isolates that required antibiotic or biocide concentrations above the MIC_50_ for inhibition. Among the resistant isolates, there were twenty-seven that exhibited MIC values ≥ the MIC for all tested biocides and antibiotics. To explore the relationship between antibiotic resistance and reduced susceptibility to biocides, a chi-square test was employed to compare the percentage of MLS-resistant isolates within two groups: those susceptible to biocides (MIC ≤ MIC_50_) and those tolerant to biocides (MIC ≥ MIC_50_) among the isolates displaying MIC values ≥ the MIC_50_ for MLS antibiotics. The Chi-square analysis yielded statistically significant results for most antibiotic-resistant isolates. This indicated a substantial distinction between isolates that exhibited tolerance to biocides and those that were susceptible to biocides. In simpler terms, isolates that required higher concentrations of antibiotics (MIC ≥ MIC_50_) were also significantly inhibited by higher biocide concentrations (MIC ≥ MIC_50_), as illustrated in Fig. 5.

**Fig. 6:**
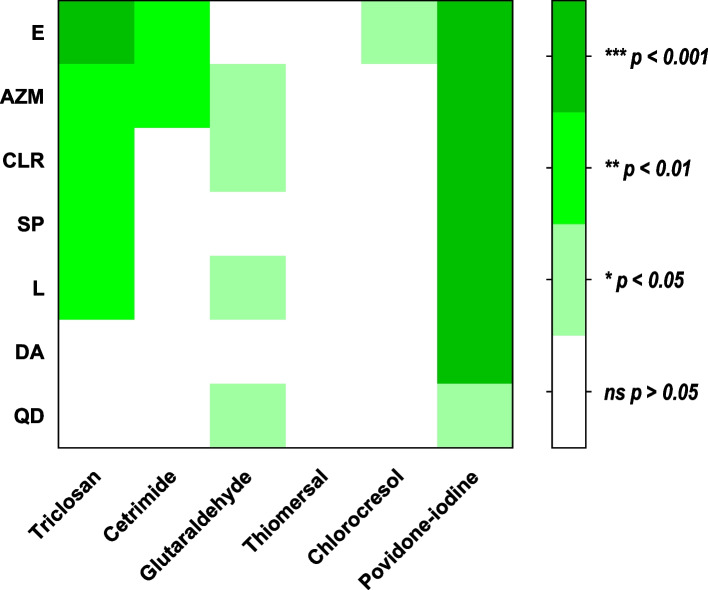
The Correlation between the resistance to MLS antibiotics and the reduced susceptibility to biocides. Pearson’s correlation coefficient of pairwise comparison was utilized to evaluate the correlation between MIC values for both biocides and antibiotics among individual isolates that exhibited MIC values ≥ MIC_50_

Furthermore, Pearson’s correlation coefficient was calculated between MIC values for biocides and MLS antibiotics for each isolate (*p* < 0.05 was considered significant) (Fig. 6). The present results showed a significant correlation between the increase in MIC values for macrolides and some biocides such as triclosan, glutaraldehyde, cetrimide, povidone-iodine, and chlorocresol. Importantly, there was a significant correlation between reduced susceptibility to povidone-iodine and resistance to all tested MLS antibiotics. On the other hand, there was no correlation between the thiomersal MIC increase and the increase of MICs of all tested MLS antibiotics.

### The MLS resistant genes distribution in the reduced susceptibility isolates with MIC ≥ MIC_50_

**Fig. 7:**
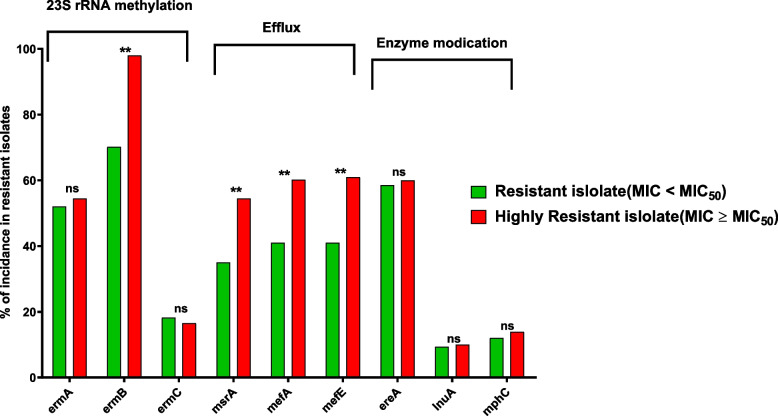
The MLS genes distribution in the tolerant isolates that showed MIC ≥ MIC_50_. The efflux encoding genes were significantly increased in the highly resistant isolates than in other resistant isolates with lower MICs < MIC_50_. This indicates the increased resistance in these isolates can be due to the augmentation of the efflux to both MLS antibiotics and biocides

To investigate the predominant resistance mechanism contributing to resistance against both biocides and MLS antibiotics, we examined the presence of MLS genes within isolates resistant to antibiotics (MIC ≥ MIC_50_) while also exhibiting reduced susceptibility to biocides (MIC ≥ MIC_50_). The genes associated with all three resistance mechanisms were detected within the isolates exhibiting high levels of resistance, in particular, *ermA*, *ermB*, *ereA*, and efflux-encoding genes *msrA*, *mefA*, and *mefE*. By employing the Chi-square test to assess the statistical difference between the incidence of MLS resistance genes in the resistant isolates (MIC ≤ MIC_50_) and the highly resistant isolates (tolerant) (MIC ≥ MIC_50_), the significant difference between resistant and tolerant isolates was observed only with *ermB* and the efflux-encoding genes (Fig. 7). The efflux-encoding genes were predominantly detected in M-phenotype isolates, which account for a significant proportion (8 out of 27 isolates) of the tolerant isolates (MIC ≥ MIC_50_). This finding highlights the critical role of efflux mechanisms in conferring resistance to both MLS antibiotics and biocides.

### Efflux assay in MLS resistant isolates

**Fig. 8:**
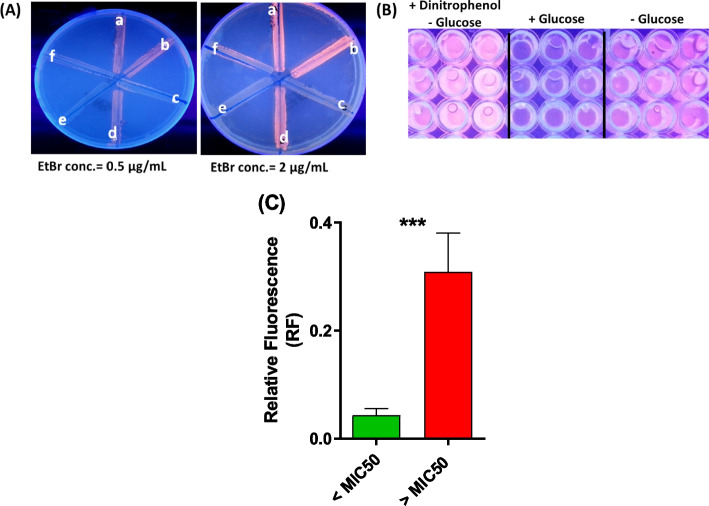
Evaluation of efflux in resistant isolates. **A** Qualitative detection of EtBr efflux by employing the EtBr agar cartwheel method. Isolates were swabbed on tryptone soya agar plates, containing different concentrations of EtBr under UV transilluminator showing positive fluorescence growth (negative efflux) (c, e), negative fluorescence growth (positive efflux) (a, b and d) and intermediate efflux (f). **B** Quantitative fluorometric assay of EtBr efflux was performed for selected resistant isolates with MICs to biocides > MIC_50_ or < MIC_50_. The efflux assays were done under conditions that grant EtBr maximum accumulation in the presence of the efflux pump inhibitor dinitrophenol and limited energy supply (absence of glucose and low temperature). **C** The EtBr efflux is presented in terms of relative fluorescence (RF). All fluorescence measurements were done at wavelengths for EtBr 530 nm for excitation and 585 nm for emission. All data were acquired at 25 °C in cycles of 60 s, for 1 h time. Each test was repeated in triplicates and the obtained results were averaged. The RF for each tested isolate (MICs to biocides > MIC_50_ or < MIC_50_) was calculated; and results were expressed as means ± SD,* p* value < 0.05 was considered significant employing Student’s *t*-test. The efflux was significantly increased in tolerant isolates with MIC ≥ MIC_50_. This indicates that the efflux plays an important role in conferring cross-resistance to both biocides and MLS antibiotics

To assess the role of the enhancement of efflux in increasing the resistance to biocides and MLS antibiotics, EtBr efflux was quantitatively assayed [39]. EtBr is a DNA-intercalating agent that fluoresces intensely upon binding to nucleic acids (e.g., within cells). In microbes with active efflux pumps, EtBr is rapidly expelled, resulting in reduced intracellular fluorescence. Conversely, in efflux-deficient cells or when efflux pumps are pharmacologically inhibited, fluorescence accumulates due to impaired efflux activity. Ten resistant isolates with lower MICs for both biocides and MLS antibiotics (MIC ≤ MIC_50_) and ten tolerant isolates (MIC ≥ MIC_50_) were chosen to quantify the efflux. The least concentrations of EtBr that produce maximum fluorescence without affecting the viability of bacterial cells ranged from 0.5 to 2 µg/ml. The EtBr efflux was assayed for each isolate in the presence or absence of glucose (0.4%) and/or DNP (325–650 µg/ml). The results were represented as relative fluorescence (RF). Each assay was conducted in triplicate, and the RF is shown as means ± standard deviation. Significantly elevated RF values were observed in isolates with MICs ≥ MIC_50_ compared to isolates with MICs ≤ MIC_50_. This finding indicates heightened efflux activity in isolates that displayed high resistance to both biocides and MLS antibiotics (Fig. 8). These findings are consistent with the observation that efflux genes *mef* and *msrA* were significantly increased in the highly resistant (tolerant) isolates compared to those with lower resistance to both biocides and antibiotics (Fig. 7).

## Discussion

Streptococci are a genus of Gram-positive bacteria responsible for a wide range of infections, from mild pharyngitis (caused by *S. pyogenes*) to severe conditions like pneumonia (*S. pneumoniae*), sepsis, and necrotizing fasciitis [27, 40]. Their clinical importance lies in their ability to cause both localized and systemic diseases, as well as post-infectious complications such as rheumatic heart disease and glomerulonephritis [41]. Treatment often involves β-lactam antibiotics like penicillin, but MLS agents are critical alternatives, especially for penicillin-allergic patients [42, 43]. MLS antibiotics inhibit bacterial protein synthesis by binding to the 50S ribosomal subunit [43]. Furthermore, lincosamides like clindamycin are valuable in deep-seated infections due to their ability to suppress toxin production and penetrate biofilms [44]. MLS antibiotics remain a key component of tailored therapy, guided by susceptibility testing and resistance patterns, ensuring effective management of streptococcal infections in diverse clinical scenarios. However, rising resistance to MLS antibiotics, driven by mechanisms such as ribosomal target modification, efflux pumps, and enzymatic inactivation, poses a significant challenge to their clinical utility [13, 20]. These resistance pathways reduce drug efficacy, complicating treatment strategies and underscoring the need for ongoing resistance surveillance to guide appropriate antibiotic stewardship.

In the current study, resistance to MLS antibiotics was screened, revealing a marked increase in resistance to all tested MLS agents. This finding aligns with previous reports [27, 45], and underscores the urgent need to investigate the underlying mechanisms driving this trend. Resistance patterns were screened, and the corresponding resistance phenotypes were characterized [27]. The resistance phenotypes are categorized mainly into four main phenotypes [27, 46, 47]. The cumulative resistance (cMLS) to the three tested antibiotics erythromycin, lincomycin, and clindamycin is widely documented and is the most prevalent phenotype [46, 47], which is in agreement with the current findings. The resistance to macrolides is more prevalent (M-phenotypes) as compared to lincomycin resistance L-phenotypes [36, 47, 48], which is in alignment with the present study.

Resistant *Streptococci* use various mechanisms to withstand MLS antibiotics, including altering the bacterial targets by methylating the 23S rRNA, employing efflux mechanisms, and producing modifying enzymes such as esterases, adenylating, and phosphorylating enzymes [13]. MLS antibiotics are different from a chemical point of view; they share the 50S ribosomal subunit binding site, resulting in stopping the translation of the bacterial protein [49]. Although several species of bacteria gain MLS resistance genes [21], MLS antibiotics anchor to different rRNA sites that could address the significant variability of the bacterial resistance to different MLS antibiotics [50]. The main resistance mechanism to MLS antibiotics is methylation of bacterial ribosomal 23S rRNA that prevents and interferes with the MLS binding to ribosomes.

The erythromycin ribosome methylase (*erm*) genes are the most predominate genes in gram-positive bacteria conferring cross-resistance to the structurally different MLS antibiotics [21, 28, 51–56]. In iMLS phenotypes, bacteria can produce inactive mRNA that remains inert until exposed to a macrolide inducer. This process enables bacteria to regulate gene expression in response to environmental cues, contributing to the development of resistance mechanisms and adaptive responses to external stimuli [13, 18, 21]. Importantly, strains that acquire *erm* genes demonstrate resistance to inducer macrolides, namely those with 14- and 15-membered rings, while maintaining susceptibility to non-inducer macrolides with 16-membered rings, as well as lincosamides and streptogramins B [46, 57, 58]. Under constitutive expression, active methylase mRNA is generated without the need for an inducer, resulting in strains that exhibit cross-resistance to MLS antibiotics [13, 18]. Moreover, resistance to macrolides and lincosamides can also arise from mutations impacting 23S rRNA ribosomal proteins L4 and L22 [48]. The prevalence of clinical isolates demonstrating constitutive resistance to MLS antibiotics is extensive, especially among methicillin-resistant strains [59]. Understanding these molecular mechanisms is essential for devising effective strategies to combat antibiotic resistance in various clinical settings. It is worthy to mention that target-site modification either by the mutation or methylation of 23S rRN could result in cross-resistance to MLS, but not to oxazolidinones [60]. There are more than thirty different *erm* genes that are inducible or constitutively expressed [18, 21]. Most of the *erm* genes are encoded on plasmids and are classified basically into four main classes: *ermA*, *B*, *C*, and *F* [18]. The current results have unveiled the identification of all examined genes within the resistant isolates. The most detected gene is *ermB* among all the isolates that were detected in all cMLS-phenotype, resistant *S. pneumoniae* and erythromycin-resistant isolates. Otherwise, *ermB* was not detected in all L-phenotypes. The least detected *erm* gene was *ermC*, which was not detected in M- and L-phenotypes and also not found in α-hemolytic *Streptococci*.

Gram-positive bacteria have the capability to resist a wide range of antibiotics through the production of drug-inactivating enzymes [61–63]. Approximately twenty genes encode enzymes such as esterases, transferases, phosphorylases, and lyases which modify and render MLS antibiotics inactive by hydrolyzing the lactone ring (*ere* genes), acetylating (*vat* genes), adenylating (*lnu* genes), or phosphorylating (*mph* genes) [21, 25]. In contrast to target modification, enzyme inactivation imparts resistance solely to structurally similar antibiotics [18], with none of these inactivating enzymes being exclusive to specific bacterial species [64]. Esterases, acetyltransferases, phosphotransferases, hydrolases, and nucleotidyl transferases have been identified in MLS resistant strains providing resistance to erythromycin and other 14- and 15-membered macrolides but not to lincosamides, known as the L phenotype [18]. The current findings showed that the *ereA* gene was the most detected gene that encodes hydrolyzing enzymes as compared to *lunA* and *mphC*. The *ereA* gene was abundantly noticed in cMLS- and L-phenotypes while *lunA* and *mphC* were only distinguished in L- and cMLS-phenotypes, respectively.

The efflux mechanism, by which *Streptococci* extrude one or more MLS antibiotics, is controlled by approximately seventeen efflux genes, employing ATP-transporters or major facilitating transporters [21, 65]. Bacterial efflux pumps are components of the cell wall, and encoded by genes are located on the bacterial chromosomes. Transferable elements are more frequently implicated in the augmented efflux of MLS [66–68]. Bacterial efflux transporters are categorized into five distinct superfamilies based on the sequence of amino acids and energy source [13, 69]. The efflux of MLS is linked partially to the cross-resistance to 14- and 15-membered macrolides and streptogramin B, primarily conferred by *msr*, *mef*, *vga*, and *isa* [21, 70]. Efflux pump compartments are inducibly expressed by erythromycin and other 14- and 15-membered macrolides [13, 21]. Clindamycin never induces or serves as an efflux pump substrate, making strains carrying the efflux genes fully susceptible to it [13]. The *mef* genes encode efflux in macrolides, while *msr* genes mediate efflux of macrolides and streptogramin B, playing a role in the active expulsion of MLS in *Streptococci* [35, 66, 71]; importantly, these genes are more commonly associated with transferable elements [66–68]. The present findings showed that the efflux-encoding genes were detected in almost all *Streptococci* spp. except *msrA*, which was not detected in *S. agalactiae*. Importantly, efflux-encoding genes were more frequently detected in the M-phenotype.

The misuse of biocides, such as using sublethal concentrations, can promote bacterial cross-resistance to antibiotics through shared resistance mechanisms like efflux pumps or genetic co-selection [72, 73]. Our observations of the widespread and improper use of biocides have led us to establish a connection between the rise in resistance to MLS antibiotics and the development of resistance to commonly used disinfectants and antiseptics. Surprisingly, a significant correlation was observed between reduced susceptibility to the most commonly used biocide, povidone-iodine, and resistance to all tested MLS antibiotics. In contrast, no correlation was found between the increase in the MIC of the least-used biocide, thiomersal, and the elevated MICs of the tested MLS antibiotics. This finding suggests that increased resistance to frequently used biocides may contribute to enhanced resistance to MLS antibiotics. To investigate the predominant resistance mechanism contributing to resistance against both biocides and MLS antibiotics, the presence of MLS resistance genes was screened in the highly resistant isolates to both antibiotics and antibiotics. The significant difference between resistant and tolerant isolates was observed only with *ermB* and the efflux-encoding genes. These findings could indicate the important role of the *ermB* gene in developing cross-resistance to biocides and MLS antibiotics. This is in agreement with the *erm* genes, in particular *ermB*, being highly transferable [21, 28, 51–55]. The efflux-encoding genes were noticed mainly in M-phenotype isolates that represent a high percentage of the tolerant isolates, which could elucidate the heightened significance of efflux mechanisms in offering resistance to both MLS antibiotics and biocides. Efflux pumps are well documented as the main mechanism behind the cross-resistance to both biocides and antibiotics, keeping in mind that the encoding genes are commonly transferable between different bacteria [22, 74].

To assess the role of the enhancement of efflux in increasing the resistance to biocides and MLS antibiotics, EtBr efflux was quantitatively assayed [39]. Significantly elevated efflux values were observed in isolates with MICs ≥ MIC_50_ (highly resistant isolates) compared to isolates with MICs ≤ MIC_50_, indicating the increased efflux activity in isolates that displayed high resistance to both biocides and MLS antibiotics. These findings are consistent with the observation that efflux genes *mef* and *msrA* were significantly increased in the highly resistant (tolerant) isolates compared to those with lower resistance to both biocides and antibiotics. These findings confirm the growing significance of efflux mechanisms in the development of cross-resistance between antibiotics and biocides, consistent with previously published data (27, 73).

## Conclusions

This study aimed to conduct a comprehensive phenotypic and genotypic characterization of resistance to MLS antibiotics among *Streptococci*. The most prevalent resistance mechanism was determined to be the target-site modification of the bacterial 50S ribosomal subunit. Constitutive resistance to MLS emerged as the most predominant phenotype, with the *erm* genes especially—*ermB* widely—distributed. The presence of genes encoding MLS inactivating enzymes was detected, notably the *ereA* gene encoding esterase, while the *lnuA* gene associated with lincomycin resistance was the least detected. The isolates exhibited varying resistance levels, with higher rates observed for macrolides and lincomycin, representing the MLS or M phenotypes, and the least resistance seen against clindamycin. A significant correlation was established between the reduced susceptibility of isolates to commonly used biocides and their resistance to MLS antibiotics. Notably, both phenotypic and genotypic evidence of increased efflux was observed in the MLS-resistant isolates, indicating a potential link between efflux and resistance to both antibiotics and biocides. However, the most involved mechanism that confers cross-resistance to both biocides and antibiotics is targeting the 50 s sub-ribosomal unit via transferable *ermB*.

## Supplementary Information

Additional file 1. Tables S1-S7. Table S1– Source of isolates. Table S2– Percentages of MLS resistance among tested streptococci. Table S3– Statistical results of the generalized mixed model. Table S4– MLS resistance patterns. Table S5– MLS genotypes. Table S6– MIC range, MIC_50_, and MIC_90_of MLS antibiotics. Table S7– MIC range, MIC_50_, and MIC_90_of biocides

## Data Availability

No datasets were generated or analysed during the current study.

## Abbreviations

MLS: Macrolides, lincosamides, and streptogramins
MICs: Minimum inhibitory concentrations
GAS: Group A streptococci
CLSI: Clinical and Laboratory Standards Institute
TSB: Tryptone soya broth
MH: Müeller-Hinton media
cMLS: Constitutive resistance phenotype
iMLS: Inducible resistance phenotype
L-phenotype: Lincosamide resistance phenotype
EtBr: Ethidium bromide
DNP: Di-nitro phenol
PBS: Phosphate buffer saline
RF: Relative fluorescence
E: Erythromycin
CLR: Clarithromycin
AZM: Azithromycin
L: Lincomycin
SP: Spiramycin
QD: Quinupristin/dalfopristin
DA: Clindamycin
erm: Erythromycin ribosome methylase genes
