# Supplementary material for: Phenotypic and genotypic characteristics of macrolide, lacosamide, and streptogramin resistance in clinically resistant Streptococci and their correlation with reduced biocide susceptibility
Source: BMC Med. 2025 May 13;23:281. doi: 10.1186/s12916-025-04097-9 (PMC12076902; doi:10.1186/s12916-025-04097-9)
Supplement: Supplementary file 1 — Additional file 1. Tables S1-S7. Table S1– Source of isolates. Table S2– Percentages of MLS resistance among tested streptococci. Table S3– Statistical results of the generalized mixed model. Table S4– MLS resistance patterns. Table S5– MLS genotypes. Table S6– MIC range, MIC50, and MIC90of MLS antibiotics. Table S7– MIC range, MIC50, and MIC90of biocides [file 12916_2025_4097_MOESM1_ESM.docx]

**Supplementary Data**

**Table S1. Source of isolates**

| **Sample** | *S. pyogenes* | *S. agalactiae,* | *S. viridians* | *S. pneumoniae* |
| --- | --- | --- | --- | --- |
| Blood | 6 | 7 | 19 | -- |
| Sputum | -- | -- | -- | 6 |
| Skin and Subcutaneous tissues | 6 | -- | -- | -- |
| Ear swabs | 5 | -- | -- | 1 |
| Biopsies from wounds | 8 | -- | -- | -- |
| Fluids and aspirates | 6 | 6 | 4 | -- |
| Total | 31 | 13 | 23 | 7 |

**Table S2. Percentages of MLS resistance among tested streptococci**

| **Antibiotic** | ***S. pyogenes* (31)** | ***S. agalactiae* (13)** | ***S. viridians* (23)** | ***S. pneumonia* (7)** |
| --- | --- | --- | --- | --- |
| **E** | 21 (**67.7 %)** | 9 (**69.2 %)** | 16 (**69.6 %)** | 7 (**100 %)** |
|  | **53 (71.6 %)** | | | |
| **AZM** | 18 (**58.1 %)** | 6 (**46.2 %)** | 12 (**52.2 %)** | 6 (**85.7 %)** |
|  | **42 (56.8 %)** | | | |
| **CLR** | 16 (**51.6 %)** | 7 (**53.8 %)** | 13 (**56.5 %)** | 5 (**71.4%)** |
|  | **41 (55.4 %)** | | | |
| **SP** | 19 (**61.3 %)** | 7 (**53.8 %)** | 15 (**65.2 %)** | 6 (**85.7 %)** |
|  | **47 (63.5 %)** | | | |
| **L** | 20 (**64.5 %)** | 8 (**61.5 %)** | 16 (**69.6 %)** | 3 (**42.9 %)** |
|  | **47 (63.5 %)** | | | |
| **DA** | 17 (**54.8 %)** | 6 (**46.2 %)** | 10 (**43.5 %)** | 0 (**0 %)** |
|  | **33 (44.6 %)** | | | |
| **QD** | **13 (41.9 %)** | **4 (30.8 %)** | **9 (39.1** | 0 (**0 %)** |
|  | **26 (35.1 %)** | | | |

**E: Erythromycin, AZM: Azithromycin, CLR: Clarithromycin, SP: Spiramycin, L: Lincomycin, DA: Clindamycin and QD: Quinupristin/Dalfopristin**

**Table S3. Statistical results of the generalized mixed model**

| Characteristic | OR | 95% CI | p-value |
| --- | --- | --- | --- |
| Antibiotic |  |  |  |
| E | Reference | |  |
| AZM | 0.28 | 0.10, 0.74 | 0.01 |
| CLR | 0.25 | 0.09, 0.66 | 0.005 |
| DA | 0.11 | 0.04, 0.31 | <0.001 |
| L | 0.59 | 0.22, 1.63 | 0.3 |
| QD | 0.07 | 0.03, 0.19 | <0.001 |
| SP | 0.59 | 0.22, 1.63 | 0.3 |
| Microorganism |  |  |  |
| *S. pyogenes* | Reference | |  |
| *S. agalactiae* | 0.7 | 0.17, 2.95 | 0.6 |
| *S. viridians* | 0.82 | 0.25, 2.74 | 0.8 |
| *S. pneumoniae* | 0.36 | 0.07, 1.91 | 0.2 |

**E: Erythromycin, AZM: Azithromycin, CLR: Clarithromycin, SP: Spiramycin, L: Lincomycin, DA: Clindamycin and QD: Quinupristin/Dalfopristin**

**Table S4. MLS resistance patterns**

| **Pattern** | **Resistance to antibiotics** | | | | | | | ***S. pyogenes*** | | ***S. agalactiae*** | | ***S. viridians*** | | ***S. pneumoniae*** | | **Total** |
| --- | --- | --- | --- | --- | --- | --- | --- | --- | --- | --- | --- | --- | --- | --- | --- | --- |
|  | **E** | **AZM** | **CLR** | **SP** | **L** | **DA** | **QD** |  |  |  |  |  |  |  |  |  |
|  |  |  |  |  |  |  |  | **No** | **%** | **No** | **%** | **No** | **%** | **No** | **%** | **%** |
| **I** | **S** | **S** | **S** | **S** | **S** | **S** | **S** | **4** | **12.9** | **2** | **15.4** | **3** | **13** | **0** | **0** | **12.2** |
| **II** | **R** | **R** | **R** | **R** | **R** | **R** | **R** | **9** | **29** | **2** | **15.4** | **6** | **26.1** | **0** | **0** | **23** |
| **III** | **R** | **R** | **R** | **R** | **R** | **R** | **S** | **2** | **6.5** | **2** | **15.4** | **2** | **8.7** | **0** | **0** | **8.1** |
| **IV** | **R** | **R** | **S** | **S** | **R** | **R** | **R** | **2** | **6.5** | **0** | **0** | **0** | **0** | **0** | **0** | **2.7** |
| **V** | **R** | **R** | **S** | **R** | **R** | **R** | **S** | **1** | **3.2** | **0** | **0** | **0** | **0** | **0** | **0** | **1.4** |
| **VI** | **R** | **S** | **S** | **R** | **R** | **R** | **S** | **1** | **3.2** | **0** | **0** | **0** | **0** | **0** | **0** | **1.4** |
| **VII** | **R** | **R** | **R** | **R** | **R** | **S** | **S** | **2** | **6.5** | **1** | **7.7** | **2** | **8.7** | **3** | **42.9** | **10.8** |
| **VIII** | **R** | **R** | **R** | **R** | **S** | **S** | **S** | **2** | **6.5** | **1** | **7.7** | **2** | **8.7** | **2** | **28.6** | **9.5** |
| **IX** | **R** | **S** | **R** | **S** | **S** | **S** | **R** | **1** | **3.2** | **0** | **0** | **0** | **0** | **0** | **0** | **1.4** |
| **X** | **R** | **S** | **S** | **S** | **S** | **S** | **S** | **1** | **3.2** | **0** | **0** | **1** | **4.3** | **1** | **14.3** | **4.1** |
| **XI** | **R** | **S** | **R** | **S** | **R** | **R** | **R** | **0** | **0** | **1** | **7.7** | **1** | **4.3** | **0** | **0** | **2.7** |
| **XII** | **R** | **S** | **S** | **S** | **R** | **S** | **S** | **0** | **0** | **1** | **7.7** | **0** | **0** | **0** | **0** | **1.4** |
| **XIII** | **R** | **S** | **S** | **S** | **R** | **S** | **R** | **0** | **0** | **0** | **0** | **1** | **4.3** | **0** | **0** | **1.4** |
| **XIV** | **R** | **S** | **S** | **R** | **S** | **S** | **S** | **0** | **0** | **1** | **7.7** | **1** | **4.3** | **0** | **0** | **2.7** |
| **XV** | **R** | **R** | **S** | **R** | **S** | **S** | **S** | **0** | **0** | **0** | **0** | **0** | **0** | **1** | **14.3** | **1.4** |
| **XVI** | **S** | **S** | **S** | **R** | **R** | **R** | **R** | **1** | **3.2** | **1** | **7.7** | **1** | **4.3** | **0** | **0** | **4.1** |
| **XVII** | **S** | **S** | **S** | **R** | **S** | **R** | **R** | **1** | **3.2** | **0** | **0** | **0** | **0** | **0** | **0** | **1.4** |
| **XVIII** | **S** | **S** | **S** | **R** | **S** | **S** | **S** | **1** | **3.2** | **0** | **0** | **0** | **0** | **0** | **0** | **1.4** |
| **XIX** | **S** | **S** | **S** | **S** | **R** | **S** | **S** | **2** | **6.5** | **1** | **7.7** | **3** | **13** | **0** | **0** | **8.1** |
| **XX** | **S** | **S** | **S** | **S** | **R** | **S** | **R** | **1** | **3.2** | **0** | **0** | **0** | **0** | **0** | **0** | **1.4** |
| **Total** |  |  |  |  |  |  |  | **31** | **100** | **13** | **100** | **23** | **100** | **7** | **100** | **100** |

**E: Erythromycin, AZM: Azithromycin, CLR: Clarithromycin, SP: Spiramycin, L: Lincomycin, DA: Clindamycin and QD: Quinupristin/Dalfopristin**

**Table S5. MLS genotypes**

| **Phenotype** | **Resistance gene profile** | | | | | | | | | ***S. pyogenes*** | ***S. agalactiae*** | ***S. viridans*** | ***S. pneumoniae*** |
| --- | --- | --- | --- | --- | --- | --- | --- | --- | --- | --- | --- | --- | --- |
|  | ***ermA*** | ***ermB*** | ***ermC*** | ***msrA*** | ***mefA*** | ***mefE*** | ***ereA*** | ***lnuA*** | ***mphC*** |  |  |  |  |
| **cMLS** | **+** | **+** | **+** | **-** | **-** | **-** | **-** | **-** | **-** | **3** | **1** |  |  |
|  | **+** | **+** | **-** | **-** | **-** | **-** | **+** | **-** | **-** | **11** | **1** | **8** |  |
|  | **+** | **+** | **-** | **-** | **-** | **-** | **-** | **-** | **+** | **2** | **1** |  |  |
|  | **+** | **+** | **-** | **-** | **-** | **-** | **+** | **-** | **+** |  |  | **1** |  |
|  | **-** | **+** | **-** | **-** | **-** | **-** | **+** | **-** | **-** | **1** | **3** |  |  |
|  | **-** | **+** | **-** | **-** | **+** | **+** | **+** | **-** | **-** |  |  | **1** |  |
| **iMLS** | **+** | **+** | **-** | **-** | **-** | **-** | **-** | **-** | **-** | **1** |  | **3** | **3** |
|  | **+** | **+** | **-** | **-** | **-** | **-** | **-** | **-** | **-** | **1** | **1** |  |  |
|  | **-** | **+** | **+** | **-** | **-** | **-** | **+** | **-** | **-** |  | **1** |  |  |
| **M** | **-** | **+** | **-** | **+** | **-** | **-** | **-** | **-** | **-** | **1** |  | **1** | **2** |
|  | **-** | **+** | **-** | **-** | **+** | **+** | **-** | **-** | **-** | **3** | **2** | **2** | **2** |
|  | **-** | **-** | **-** | **-** | **+** | **+** | **-** | **-** | **-** | **1** |  | **1** |  |
| **L** | **-** | **-** | **-** | **-** | **-** | **-** | **+** | **-** | **-** | **1** |  |  |  |
|  | **-** | **-** | **-** | **-** | **-** | **-** | **+** | **+** | **-** | **2** | **1** | **3** |  |

**Table S6. MIC range, MIC_50_, and MIC_90_ (μg/ml) of MLS antibiotics**

| **Antibiotic** | **MIC range** | **MIC_50_** | **MIC_90_** |
| --- | --- | --- | --- |
| **E** | **0.125 - 1024** | **247** | **860** |
| **AZM** | **0.125 - 1024** | **185** | **800** |
| **CLR** | **0.125 - 1024** | **267** | **590** |
| **SP** | **0.125 - 1024** | **125** | **775** |
| **L** | **0.125 - 1024** | **52** | **650** |
| **DA** | **0.125 - 1024** | **275** | **625** |
| **QD** | **0.125 - 1024** | **32** | **725** |

**E: Erythromycin, AZM: Azithromycin, CLR: Clarithromycin, SP: Spiramycin, L: Lincomycin, DA: Clindamycin and QD: Quinupristin/Dalfopristin**

**Table S7. MIC range, MIC_50_, and MIC_90_ (μg/ml) of biocides**

| **Biocides** | **MIC range** | **MIC_50_** | **MIC_90_** |
| --- | --- | --- | --- |
| **Triclosan** | **0.5 – 6.0** | **2.2** | **5.4** |
| **Cetrimide** | **0.5 – 5.0** | **1.45** | **3.6** |
| **Glutaraldehyde** | **0.2 - 0.9** | **0.43** | **0.8** |
| **Thiomersal** | **0.5 - 3** | **1.3** | **2.6** |
| **Chlorocresol** | **150 - 600** | **280** | **570** |
| **Povidone-iodine** | **900-1500** | **1050** | **1400** |
